# Supplementary material for: Designing an irreversible metabolic switch for scalable induction of microbial chemical production
Source: Nat Commun. 2021 Jun 8;12:3419. doi: 10.1038/s41467-021-23606-x (PMC8187666; doi:10.1038/s41467-021-23606-x)
Supplement: Supplementary file 2 — Reporting Summary [file 41467_2021_23606_MOESM2_ESM.pdf]

nature research

Corresponding author(s): Professor Declan Gerard Bates

Last updated by author(s): Apr 30, 2021

Reporting Summary

Nature Research wishes to improve the reproducibility of the work that we publish. This form provides structure for consistency and transparency in reporting. For further information on Nature Research policies, see our Editorial Policies and the Editorial Policy Checklist.

Statistics

For all statistical analyses, confirm that the following items are present in the figure legend, table legend, main text, or Methods section.

n/a	Confirmed	
☐	☒	The exact sample size (n) for each experimental group/condition, given as a discrete number and unit of measurement
☒	☐	A statement on whether measurements were taken from distinct samples or whether the same sample was measured repeatedly
☒	☐	The statistical test(s) used AND whether they are one- or two-sided<br>*Only common tests should be described solely by name; describe more complex techniques in the Methods section.*
☒	☐	A description of all covariates tested
☒	☐	A description of any assumptions or corrections, such as tests of normality and adjustment for multiple comparisons
☐	☒	A full description of the statistical parameters including central tendency (e.g. means) or other basic estimates (e.g. regression coefficient) AND variation (e.g. standard deviation) or associated estimates of uncertainty (e.g. confidence intervals)
☒	☐	For null hypothesis testing, the test statistic (e.g. F, t, r) with confidence intervals, effect sizes, degrees of freedom and P value noted<br>*Give P values as exact values whenever suitable.*
☒	☐	For Bayesian analysis, information on the choice of priors and Markov chain Monte Carlo settings
☒	☐	For hierarchical and complex designs, identification of the appropriate level for tests and full reporting of outcomes
☒	☐	Estimates of effect sizes (e.g. Cohen's d, Pearson's r), indicating how they were calculated

Our web collection on statistics for biologists contains articles on many of the points above.

Software and code

Policy information about availability of computer code

Data collection	Where data could not be collected from the published source that data was regenerated from a snapshot of the data plotted in the source paper using web application WebPlotDigitizer (version: 4.4, date: November 2020, author: Ankit Rohatgi, website: https://automeris.io/WebPlotDigitizer).
Data analysis	All computational models, data analysis and generation were conducted in MATLAB R2018a, and .m scripts of the model and analyses to replicate results in the paper and supplementary information can be made available from the corresponding author upon request.

For manuscripts utilizing custom algorithms or software that are central to the research but not yet described in published literature, software must be made available to editors and reviewers. We strongly encourage code deposition in a community repository (e.g. GitHub). See the Nature Research guidelines for submitting code & software for further information.

Data

Policy information about availability of data

All manuscripts must include a data availability statement. This statement should provide the following information, where applicable:

- Accession codes, unique identifiers, or web links for publicly available datasets
- A list of figures that have associated raw data
- A description of any restrictions on data availability

Example nutrient inducible uptake systems reported in Supplementary Table 1 were sourced from EcoCyc. All relevant data generated and analysed in this study are included within the paper and its Supplementary Information. Also, data used from published sources, including those used for model parameterization, are referenced within the paper and the Supplementary Information. Additional source data beyond those presented in this paper and Supplementary Information are available from the corresponding author upon request.

Field-specific reporting

Please select the one below that is the best fit for your research. If you are not sure, read the appropriate sections before making your selection.

- [x] Life sciences
- [ ] Behavioural & social sciences
- [ ] Ecological, evolutionary & environmental sciences

For a reference copy of the document with all sections, see nature.com/documents/nr-reporting-summary-flat.pdf

Life sciences study design

All studies must disclose on these points even when the disclosure is negative.

Sample size	We used the eFAST method for global sensitivity analysis and sampled 300 random parameter sets to calculate the first-order sensitivity indices (100 samples, done in triplicate), and report their average and standard deviation values in Supplementary Figure 2.
Data exclusions	No data was excluded from the analyses performed throughout the study.
Replication	We used the eFAST method for global sensitivity analysis, but given the stochastic nature of the algorithm we repeated calculation of the first-order sensitivity indices in triplicate, and report their average and standard deviation values in Supplementary Figure 2. For published data used in model parameterization, we reported the number of biological replicates made to generate that data. All other data in the study was generated from mathematical models, which can be reproduced and so there was no need for replication.
Randomization	No clinical data was used and so randomization of data was not applicable.
Blinding	No clinical data was used in this study and so blinding of data samples was not applicable.

Reporting for specific materials, systems and methods

We require information from authors about some types of materials, experimental systems and methods used in many studies. Here, indicate whether each material, system or method listed is relevant to your study. If you are not sure if a list item applies to your research, read the appropriate section before selecting a response.

Materials & experimental systems

n/a	Involved in the study	
x		Antibodies
x		Eukaryotic cell lines
x		Palaeontology and archaeology
x		Animals and other organisms
x		Human research participants
x		Clinical data
x		Dual use research of concern

Methods

n/a	Involved in the study	
x		ChIP-seq
x		Flow cytometry
x		MRI-based neuroimaging
